# The role of phosphoprotein phosphatases catalytic subunit genes in pancreatic cancer

**DOI:** 10.1042/BSR20203282

**Published:** 2021-01-04

**Authors:** Junjie Hang, Steven Yuk-Fai Lau, Ruohan Yin, Lina Zhu, Siyuan Zhou, Xin Yuan, Lixia Wu

**Affiliations:** 1 Changzhou No. 2 People’s Hospital, Nanjing Medical University, Xinglong Road 19, Changzhou 213000, China; 2 Division of Biostatistics, JC School of Public Health and Primary Care, The Chinese University of Hong Kong, Prince of Wales Hospital, Shatin, New Territories, Hong Kong, China; 3 Department of Oncology, Shanghai JingAn District ZhaBei Central Hospital, Zhonghuaxin Road 619, Shanghai 200040, China

**Keywords:** biological function, pancreatic cancer, phosphoprotein phosphatases catalytic subunit, prognostic value

## Abstract

Compelling evidence suggests that phosphoprotein phosphatases (PPPs) are involved in a large spectrum of physiological and pathological processes, but little is known about their roles in pancreatic cancer. We investigated the expression level, prognostic value, and potential function of PPPs with data from Oncomine, GEPIA, THPA, and TCGA databases and an independent cohort of patients with pancreatic cancer. Among all the PPP catalytic subunits (PPPcs), the transcription levels of PPP1CA, PPP1CB, PPP3CA, PPP3CB, and PPP4C were higher in pancreatic cancer than in normal pancreas (*P*<0.01, fold change > 2). Kaplan–Meier analysis showed that high transcription levels of PPP1CA, PPP1CB, PPP2CA, PPP2CB, PPP3CA, and PPP4C correlated with poorer survival. In contrast, patients with high levels of PPP3CB, PPP3CC, PPP5C, PPP6C, and PPEF2 had much better prognoses. Data from THPA and patients with pancreatic cancer enrolled in our hospital also confirmed the prognostic value of PPP1CA, PPP1CB, PPP2CA, PPP2CB, PPP3CA, PPP3CB, and PPP6C at the protein level. In addition, the Pearson Chi-square test showed that PPP3CB level was significantly correlated with T and N stages. GO and KEGG analyses showed that the genes and pathways related to the pathogenesis and progression of pancreatic cancer were greatly affected by alterations in PPPcs. Results of the present study suggest that PPP1CA, PPP1CB, PPP2CA, PPP2CB, and PPP3CA have deleterious effects but PPP3CB, PPP5C, and PPP6C have beneficial effects on pancreatic cancer.

## Introduction

Pancreatic cancer is a highly lethal malignancy, with almost as many cancer-related deaths (*n*=432,242) as cases (*n*=459,000) worldwide [1]. In China, there were an estimated 90,100 new cases and 79,400 deaths as a result of pancreatic cancer in 2015 [2]. Because of limited progress in diagnostic methods and effective therapeutic interventions, the prognosis for patients with pancreatic cancer remains dismal [3,4]. Although a small portion of patients experience long-term survival of more than 10 years, the 5-year overall survival rate is approximately 6% [5]. Thus, there is an urgent need to identify valuable biomarkers and promising therapeutic targets to improve the current diagnostic and treatment strategy for pancreatic cancer [6].

The phosphoprotein phosphatases (PPPs), which belong to the protein phosphatase family, consist of Ppp1 to Ppp7 [7]. PPPs are involved in a variety of physiological and pathological processes, such as mitosis, gene expression, and cancer [8]. The PPP family consists of conserved catalytic subunits, including PPP1C to PPP7C (also known as PPEF), which diversify their biological functions by associating with different noncatalytic subunits [9]. The PPP catalytic subunits (PPPcs) have 13 different isoforms: PPP1CA, PPP1CB, PPP1CC, PPP2CA, PPP2CB, PPP3CA, PPP3CB, PPP3CC, PPP4C, PPP5C, PPP6C, PPEF1, and PPEF2 (Supplementary Table S1); even in the same PPPcs, different isoforms boast differential biochemical and regulatory properties [10].

Among all these PPPcs, only PPP6C was explicitly identified as an oncogene in previous reports [11], although other PPPs or PPPcs also showed important roles in the pathogenesis and progression of pancreatic cancer. For example, gemcitabine induced oxidative stress in pancreatic cancer cells, with an increase in p-eukaryotic initiation factor 2 (p-eIF2) levels, and this process could be attenuated by PP1-mediated p-eIF2 dephosphorylation [12]. Farrell et al. showed that the knockdown of an endogenous and cancerous inhibitor of PP2A could increase c-Myc degradation and decrease the tumorigenic potential of pancreatic cancer cells [13]. In addition, Tahira et al. found that the long noncoding intronic RNA of PPP3CB was differentially expressed in primary and metastatic pancreatic cancer, but the mechanism remains unelucidated [14]. Although compelling evidence suggests that PPPs or PPPcs are involved in various cancers, little is known about their roles in pancreatic cancer. The involvement of PPPcs in the regulation of a large spectrum of physiological processes and the growing interest in phosphatases as drug targets have increased our interest in investigating the expression level, prognostic value, and potential biological function of PPPs in pancreatic cancer.

## Materials and methods

### Oncomine database

Oncomine is a web-based database (www.oncomine.org) that boasts a variety of public cancer microarray data for mining. The mRNA expression levels of PPPcs were compared between cancerous and normal tissues in different datasets. A two-sided *t*-test was used for this differential expression analysis. The optimal *P*-value and fold change were determined to be 0.01 and 2 [15].

### GEPIA

The transcription levels of PPPcs as well as the correlation between PPPcs and tumor stage for pancreatic cancer were investigated by GEPIA (Gene Expression Profiling Interactive Analysis). GEPIA, a web-based server (http://gepia.cancer-pku.cn/) for cancer and normal gene expression profiling, provides interactive functions, including correlation analysis and differential expression analysis, of content from the TCGA and GTEx datasets [16].

### The Human Protein Atlas

The Human Protein Atlas (www.proteinatlas.org) is a Swedish-based program of protein expression data based on 17,000 genes and approximately 26,000 antibodies. THPA is composed of three parts: the tissue atlas, cell atlas, and pathology atlas. We used THPA to investigate the protein levels of PPPcs in both the normal pancreas and pancreatic cancer. In addition, the impact of PPPcs on survival of patients with pancreatic cancer was evaluated with THPA; *P*<0.001 was considered statistically significant [17].

### cBioPortal

The cBioPortal website (https://www.cbioportal.org/) provides an online platform to integrate, explore, and analyze cancer genomics data from various resources, such as The Cancer Genome Atlas (TCGA). We used data from 186 patients with pancreatic cancer included in TCGA dataset (Provisional) to evaluate the genomic alterations, expression correlations, and network of PPPcs with cBioPortal [18].

### DAVID

DAVID bioinformatics resources consist of a variety of publicly available tools for the functional analysis of large gene lists. PPPcs and the 50 neighbors with the highest alteration frequency were submitted to DAVID for additional functional annotation, such as Gene Ontology (GO) and Kyoto Encyclopedia of Genes and Genomes (KEGG). In this work, we mainly focused on three aspects of GO analysis: biological processes (BP), cellular components (CC), and molecular functions (MF). In addition, we used KEGG analysis to investigate the pathways in which PPPcs and their neighbor genes were involved [19].

### Patients and specimens

A total of 55 patients with pancreatic cancer were enrolled at the Changzhou No. 2 People’s Hospital. Inclusion criteria were as follows: (1) pathologically confirmed pancreatic ductal adenocarcinoma, either by surgical resection or needle biopsy; (2) no concurrent cancer at another organ site; and (3) available clinical data at the time of first diagnosis. Cancerous tissue was collected during surgery or biopsy and was histopathologically confirmed. We also collected clinicopathological information from patients, including age, sex, primary tumor location, nuclear grade, vascular invasion, TNM stage, and clinical manifestation (Supplementary Table S2). Patients’ written informed consents and approval from the Ethics Committee of Changzhou No. 2 People’s Hospital were obtained for the use of these clinical materials. Tissue microarray of these specimens was constructed as previously described. Briefly, representative tumor regions were defined as areas containing >75% cancer cells. Tissue cylinders (1.5 mm in diameter) were punched from the defined regions of the block using a tissue microarray (Century, IL, CA, U.S.A.) and deposited in a paraffin block. Sections of the resulting tissue microarray blocks were transferred to glass slides.

### Immunohistochemistry and evaluation

The tissue microarrays were dewaxed and dehydrated in xylene and an alcohol bath solution. Antigen retrieval was performed by setting the slides in 0.01 M sodium citrate (pH 6.0) at 98°C for 5 min. After that, endogenous peroxidase activity was immersed in 3% hydrogen peroxide for 10 min. The slides were cooled to room temperature and blocked by incubation with normal goat serum at room temperature for 30 min, followed by incubation with the mouse monoclonal antibody against PPP3CB (sc-365612, Santa Ceruz) at 4°C overnight. Then, the sections were incubated by appropriate biotin-labeled secondary antibody and positive signals were developed in a 3, 3′-diaminobenzidine tetrahydrochloride (DAB) solution.

PPP3CB immunostaining signals were evaluated by two researchers, with the clinical information blinded to them, and scored. Brown cytoplasmic staining for PPP3CB was considered positive. The percentage of PPP3CB-positive cells was scored with the following four categories: 1 (<25%), 2 (25–50%), 3 (50–75%), and 4 (>75%). The staining intensity of positive cells was scored as 0 (absent), 1 (weak infiltration), 2 (moderate infiltration), and 3 (strong infiltration). The final score was the sum of the intensity and the percentage.

### Statistical analysis

The statistical analyses were performed with SPSS statistical software (version 21.0, SPSS Inc, IBM, Armonk, NY, U.S.A.). The optimal cutoff value of PPPcs was determined on the basis of overall survival (OS) by using an X-tile plot (version 3.6.1) [20]. The correlations between the expression of PPPcs and clinicopathological variables were assessed with the Pearson Chi-square test. Kaplan–Meier analysis was performed to assess the prognostic value of PPPcs. A multivariate Cox regression model was used to select prognostic variables from all PPPcs. Then, a prognostic score (PS) was constructed with these variables, as follows: PS = 0.50 × PPP1CB + 0.67 × PPP3CA − 0.53 × PPP3CB − 0.51 × PPEF2. In this formula, each PPPcs was weighted by its β-coefficient derived by the Cox regression model. The area under the curve (AUC) was used to assess the predictive accuracy of the score. A two-sided *P*<0.05 was considered statistically significant in all tests.

## Results

### Transcription levels of PPPcs in patients with pancreatic cancer

Thirteen PPPcs genes were included in the analysis. The transcription levels of PPPcs were compared between pancreatic cancer and normal tissues with Oncomine databases. PPP4C was the most significantly up-regulated gene in pancreatic cancer, with a fold change of 6.153 in the dataset from Grutzmann, and was upregulated in the dataset from Logsdon and Pei [21–23]. Logsdon demonstrated that the mRNA expression level of PPP3CB had a fold change of 3.002 in patients with pancreatic adenocarcinoma compared with normal tissues [22]. In addition, the dataset from Segara and Bagea showed that the levels of PPP3CA in pancreatic adenocarcinoma were higher than those in normal tissues; their fold changes were 2.564 and 2.077, respectively [24,25]. A trend was seen in PPP1CA, PPP1CB, and PPEF1: they were also overexpressed in pancreatic cancer, with fold changes between 1.5 and 2.0 [23,24,26]. However, no significant difference was observed in the expression of PPP1CC, PPP2CA, PPP2CB, PPP3CC, PPP5C, PPP6C, and PPEF2 between pancreatic adenocarcinoma and normal pancreatic tissue (Table 1).

**Table 1.** Significant changes of PPPs expression in transcription level between pancreatic cancer and normal pancreas

Genes	Isoforms	Pancreatic cancer versus pancreas	Fold change	*P*-value	*t*-test	Reference
PPP1C	PPP1CA	Pancreatic adenocarcinoma versus Normal	1.786	2.47E-05	5.633	Iacobuzio [27]
		Pancreatic adenocarcinoma versus Normal	1.847	6.39E-05	4.636	Pei [24]
	PPP1CB	Pancreatic adenocarcinoma versus Normal	1.985	0.001	3.724	Segara [25]
	PPP1CC	NA	NA	NA	NA	NA
PPP2C	PPP2CA	NA	NA	NA	NA	NA
	PPP2CB	NA	NA	NA	NA	NA
PPP3C	PPP3CA	Pancreatic adenocarcinoma versus Normal	2.564	6.95E-04	3.911	Segara [25]
		Pancreatic adenocarcinoma versus Normal	2.077	1.74E-09	7.233	Badea [26]
	PPP3CB	Pancreatic adenocarcinoma versus Normal	3.267	0.001	4.739	Logsdon [23]
	PPP3CC	NA	NA	NA	NA	NA
PPP4C		Pancreatic adenocarcinoma versus Normal	1.871	6.76E-05	5.417	Logsdon [23]
		Pancreatic adenocarcinoma versus Normal	6.153	0.005	2.877	Grutzmann [22]
		Pancreatic carcinoma versus Normal	1.730	7.26E-07	6.048	Pei [24]
PPP5C		NA	NA	NA	NA	NA
PPP6C		NA	NA	NA	NA	NA
PPP7C	PPEF1	Pancreatic carcinoma versus Normal	1.642	2.50E-05	4.439	Pei [24]
	PPEF2	NA	NA	NA	NA	NA

Abbreviation: NA, no association.

**Figure 1:**
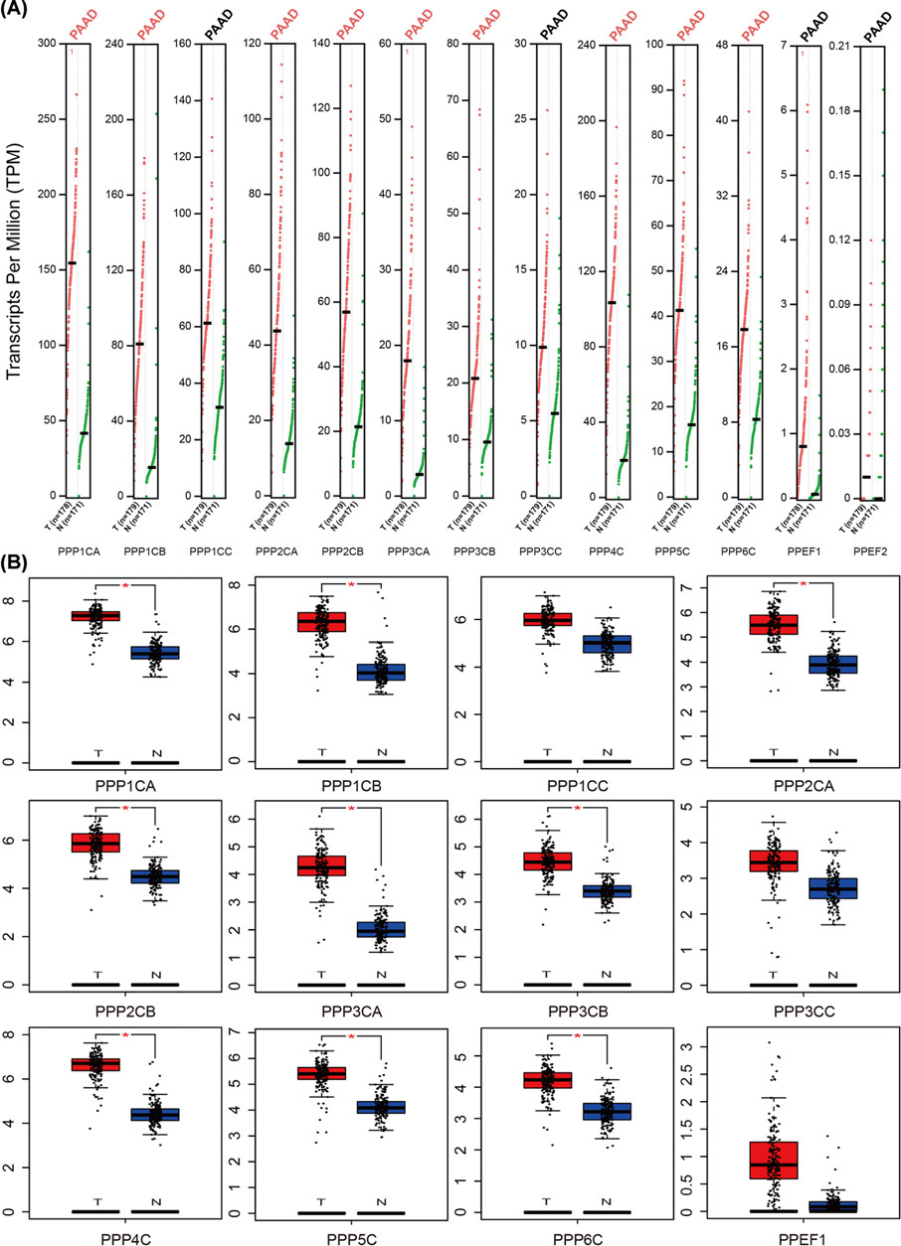
The transcription levels of PPPcs in pancreatic cancer The expression profile of PPPcs in pancreatic cancer and normal pancreas tissues in GEPIA datasets (**A**) and the box plots with jitter for comparing them (**B**). PPPcs with *P*<0.01 are considered differentially expressed genes (red). Conversely, PPPcs with *P*≥0.01 are considered not differentially expressed genes (black).

We also used GEPIA datasets to confirm the results (Figure 1A). We found that the mRNA expression levels of PPP1CA, PPP1CB, PPP2CA, PPP2CB, PPP3CA, PPP3CB, PPP4C, PPP5C, and PPP6C were higher in pancreatic adenocarcinoma than in normal tissues. Likewise, there was no significant difference in the mRNA levels of PPP1CC, PPP3CC, and PPEF2 between pancreatic adenocarcinoma and normal pancreas. In addition, PPEF1 showed low expression in both pancreatic adenocarcinoma and normal tissues (Figure 1B).

### Correlations between PPPcs and clinicopathological variables

We investigated the correlations between transcription level of PPPcs and clinicopathological variables with the Pearson Chi-square test. Patients with pancreatic cancer from TCGA dataset were divided into two groups according to age (≥60 or <60 years), T stage (1/2 or 3/4), and N state (0 or 1). Then, the mRNA level of PPPcs, also divided into two groups with the optimal cutoff value determined by the X-tile plot, were compared between these groups (Table 2). There was a positive correlation between PPP3CB and T stage (*P*=0.025). PPP5C levels were significantly correlated with both T stage and N stage (*P*<0.05). In addition, a statistically significant association of PPP4C was observed with age (*P*=0.047). We also confirmed the correlations between PPPs and tumor stages with GEPIA. Only the PPP3CB and PPP5C levels significantly varied by stage, whereas levels of other PPPcs groups did not show any significant differences at different stages (Supplementary Figure S1).

**Table 2:** Comparison of baseline characteristics according to PPPcs

Characteristics	Age		*P*-value	T		*P*-value	N		*P*-value
	<60	≥60		1 or 2	3 or 4		0	1	
PPP1CA									
Low	30	58	0.25	20	68	0.075	27	61	0.741
High	23	65		11	77		25	63	
PPP1CB									
Low	26	76	0.116	15	87	0.234	34	68	0.196
High	27	47		16	58		18	56	
PPP1CC									
Low	38	86	0.812	22	102	0.945	33	91	0.188
High	15	37		9	43		19	33	
PPP2CA									
Low	25	71	0.197	21	75	0.104	28	68	0.904
High	28	52		10	70		24	56	
PPP2CB									
Low	17	42	0.789	14	45	0.13	19	40	0.583
High	36	81		17	100		33	84	
PPP3CA									
Low	41	105	0.195	29	117	0.084	45	101	0.413
High	12	18		2	28		7	23	
PPP3CB									
Low	24	65	0.357	10	79	0.025^*^	24	65	0.448
High	29	58		21	66		28	59	
PPP3CC									
Low	8	11	0.228	2	17	0.391	5	14	0.744
High	45	112		29	128		47	110	
PPP4C									
Low	27	43	0.047^*^	17	53	0.059	22	48	0.656
High	26	80		14	92		30	76	
PPP5C									
Low	48	111	0.947	24	135	0.019^*^	43	116	0.026^*^
High	5	12		7	10		9	8	
PPP6C									
Low	33	90	0.148	19	104	0.25	32	91	0.118
High	20	33		12	41		20	33	
PPEF1									
Low	20	52	0.574	15	57	0.351	25	47	0.21
High	33	71		16	88		27	77	
PPEF2									
Low	29	74	0.501	15	88	0.207	27	76	0.25
High	24	49		16	57		25	48	

* *P*<0.05

### Correlations between PPPcs and prognosis

The correlations between the PPPcs family and the prognosis of patients with pancreatic cancer from TCGA dataset were investigated by Kaplan–Meier analysis. The results demonstrated that patients with higher transcriptional levels of PPP1CA, PPP1CB, PPP2CA, PPP2CB, PPP3CA, and PPP4C showed poorer prognoses than those with lower levels. Conversely, patients with higher transcriptional levels of PPP3CB, PPP3CC, PPP5C, PPP6C, and PPEF2 had better OS than those with lower levels (*P*<0.05). However, there was no significant difference in OS between groups with different levels of PPP1CC and PPEF1 (Supplementary Figure S2). A multivariate Cox regression model was used to select prognostic variables from all PPPcs for patients with pancreatic cancer from TCGA dataset. Four PPPcs PPP1CB, PPP3CA, PPP3CB, and PPEF2 were selected to construct a prognostic score (PS), in which PS = 0.50 × PPP1CB + 0.67 × PPP3CA − 0.53 × PPP3CB − 0.51 × PPEF2. In this formula, low expression status was equivalent to 0, and high expression status was equivalent to 1. This model showed an acceptable C-index of 0.765 (Supplementary Figure S3).

### The protein expression of PPPcs in pancreatic cancer

**Figure 2:**
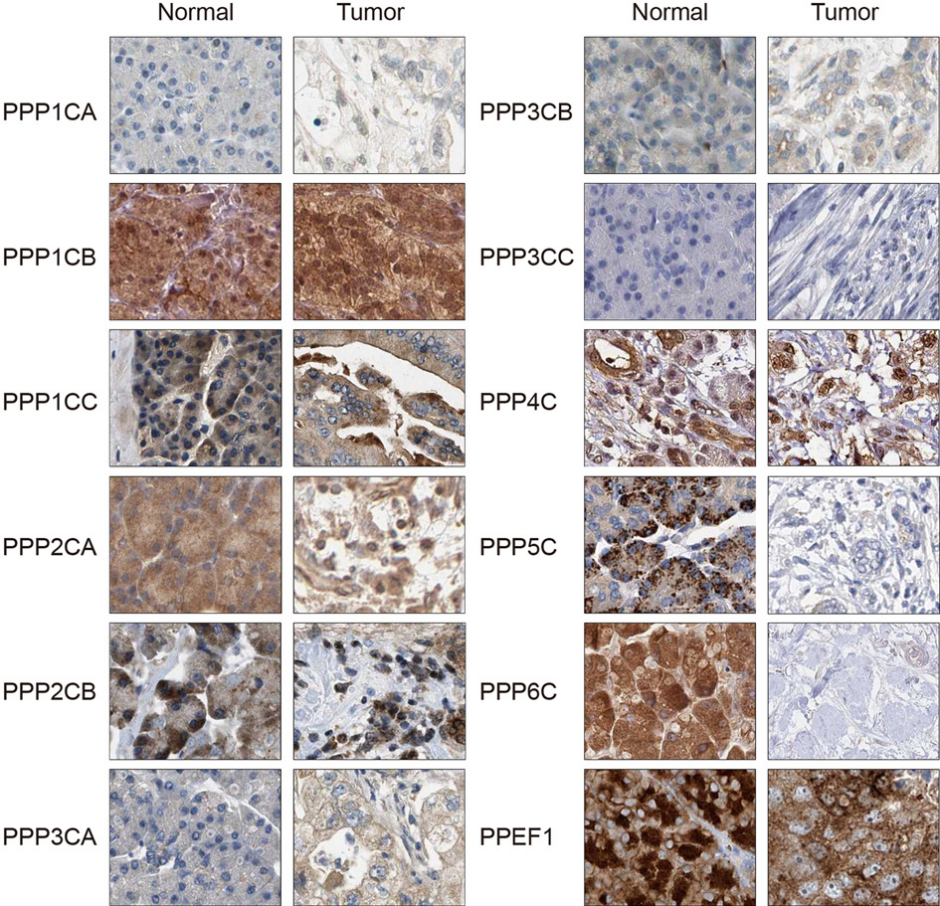
The protein levels of PPPcs in pancreatic cancer in THPA

We investigated the protein expression of PPPcs in pancreatic cancer tissues and in normal pancreatic tissues with THPA. We found that PPP1CA and PPP3CA were more highly expressed in the cancerous tissues than in the normal pancreatic tissues. In contrast, THPA showed that the expression of PPP5C and PPP6C was higher in the normal pancreatic tissues than in cancerous tissues. There was no significant difference in the expressions of PPP1CB, PPP1CC, PPP2CA, PPP2CB, PPP3CB, PPP3CC, PPP4C, and PPEF1 between cancerous and normal pancreatic tissues (Figure 2). In addition, only PPP3CB and PPP6C showed favorable prognostic values with regard to protein level, whereas PPP1CA, PPP1CB, PPP1CC, PPP2CA, PPP2CB, and PPP3CA showed unfavorable prognostic values in pancreatic cancer (*P*<0.05, Supplementary Table S3).

PPP3CB showed the most significant prognostic value, with the smallest *P*-value of 0.0000696, so we further validated this result by immunohistochemistry on pancreatic cancer tissue from an independent cohort of patients. The representative images of PPP3CB and the distribution pattern of the immunohistochemical results are shown in Supplementary Figure S4. The Pearson Chi-square test showed that the expression of PPP3CB was higher in patients with nuclear grade I to II cancer than those with nuclear grade III cancer (*P*=0.075). In addition, as shown in Table 3, the expression of PPP3CB correlated with T stage (*P*=0.015) and N stage (*P*=0.007). In the univariate analysis, nuclear grade (*P*=0.021), N stage (*P*<0.001), and PPP3CB (*P*<0.001) significantly correlated with OS (Table 4). These variables were assessed in multivariate analysis, and all of them demonstrated independent prognostic values (*P*=0.032 for nuclear grade, *P*=0.009 for N stage, and *P*<0.001 for PPP3CB).

**Table 3:** Correlations between PPP3CB expression and clinicopathological characteristics

	PPP3CB low expression (*n*=23)	PPP3CB high expression (*n*=32)	*P*-value
Age			
<60	10 (43.5%)	12 (37.5%)	0.655
≥60	13 (56.5%)	20 (62.5%)	
Sex			
Male	15 (65.2%)	22 (68.8%)	0.783
Female	8 (34.8%)	10 (31.3%)	
Primary tumor location			
Head and neck	14 (60.9%)	18 (56.3%)	0.732
Body and tail	9 (39.1%)	14 (43.8%)	
Nuclear grade			
I-II	16 (69.6%)	29 (90.6%)	0.075
III	7 (30.4%)	3 (9.4%)	
Vascular invasion			
No	13 (56.5%)	15 (46.9%)	0.480
Yes	10 (43.5%)	17 (53.1%)	
T stage			
1-2	23 (100.0%)	24 (75.0%)	0.015
3	0 (0%)	8 (25.0%)	
N stage			
0	9 (39.1%)	24 (75.0%)	0.007
1	14 (60.9%)	8 (25.0%)	
Jaundice			
No	17 (73.9%)	25 (78.1%)	0.717
Yes	6 (26.1%)	7 (21.9%)	
Abdominal pain			
No	10 (43.5%)	12 (37.5%)	0.655
Yes	13 (56.5%)	20 (62.5%)	

**Table 4:** Univariate and multivariate survival analysis of clinicopathological variables in patients with pancreatic cancer

Factors	Univariate analysis			Multivariate analysis		
	HR	95%CI	*P*-value	HR	95%CI	*P*-value
Age						
≥60	Ref					
<60	1.292	0.689–2.422	0.425			
Sex						
Male	Ref					
Female	0.549	0.269–1.123	0.101			
Primary tumor location						
Head and neck	Ref					
Body and tail	0.971	0.521–1.810	0.926			
Nuclear grade						
I-II	Ref			Ref		
III	2.421	1.146–5.117	0.021	2.399	1.077–5.347	0.032
Vascular invasion						
No	Ref					
Yes	1.578	0.848–2.939	0.150			
T stage						
1-2	Ref					
3	0.704	0.276–1.798	0.464			
N stage						
0	Ref			Ref		
1	3.453	1.803–6.611	<0.001	2.583	1.269–5.257	0.009
Jaundice						
No	Ref					
Yes	1.135	0.556–2.316	0.728			
Abdominal pain						
No	Ref					
Yes	0.765	0.412–1.421	0.396			
PPP3CB						
Low expression	Ref			Ref		
High expression	0.114	0.052–0.248	<0.001	0.164	0.075–0.360	<0.001

### Potential functions of PPPcs in pancreatic cancer

**Figure 3:**
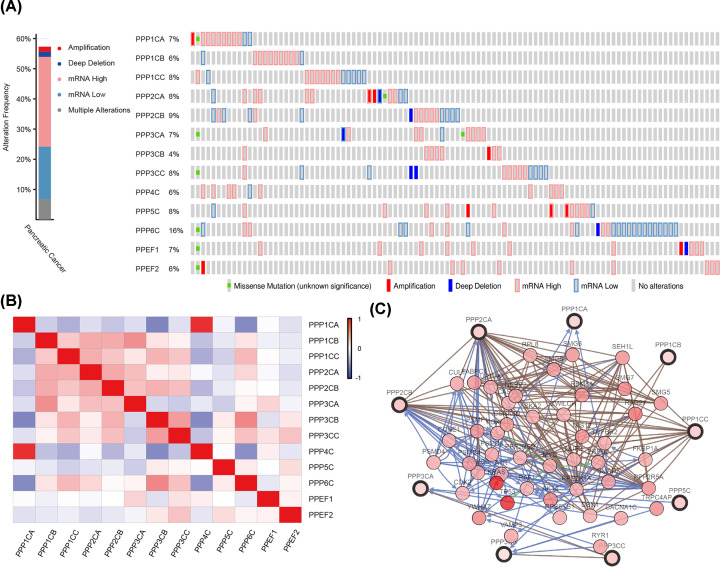
The mutation analysis, correlation analysis, and network analysis of PPPcs in cBioPortal The mutation analysis (**A**), correlation analysis (**B**), and network analysis (**C**) of PPPcs.

The genomic alterations, expression correlations, and network of genes in the PPPcs family were investigated with the online tool, cBioPortal, for pancreatic adenocarcinoma. The genomic alterations of PPPcs were observed in 102 samples (57.3%) of 178 patients (Figure 3A). The mutual exclusivity and co-occurrence analysis of genomic alterations in each pair of PPPcs showed that only three gene pairs had the tendency toward co-occurrence. The pairs included PPP1CA with PPP4C (*P*=0.005), PPP2CB with PPP3CC (*P*=0.015), and PPP3CA with PPEF1 (*P*=0.036). Using the Pearson’s correlation, we calculated the association between PPPcs in mRNA level (RNA Seq V2 RSEM). Positive correlations were found in the following pairs: PPP1CA with PPP3CB (*r* =−0.45), PPP1CA with PPP4C (*r* = 0.83), PPP1CB with PPP3CA (*r* = 0.42), PPP3CB with PPP4C (*r* =−0.44), PPP3CB with PPP6C (*r* = 0.46), and PPP4C with PPP6C (*r* =−0.42) (Figure 3B). Analysis of the networks of PPPcs genes and the 50 neighbors with the highest alteration frequencies was conducted with cBioPortal. The results showed that the genes related to the pathogenesis and progression of pancreatic cancer, such as AKT2, TGFBR2, RAF1, TP53, and ERBB2, would be greatly affected by alterations in PPPcs. Among all the PPPcs, PPP1CB, PPP1CC, PPP2CA, PPP2CB, PPP3CB, and PPP5C had tight associations with at least one of the pancreatic cancer related genes in that list (Figure 3C).

**Figure 4:**
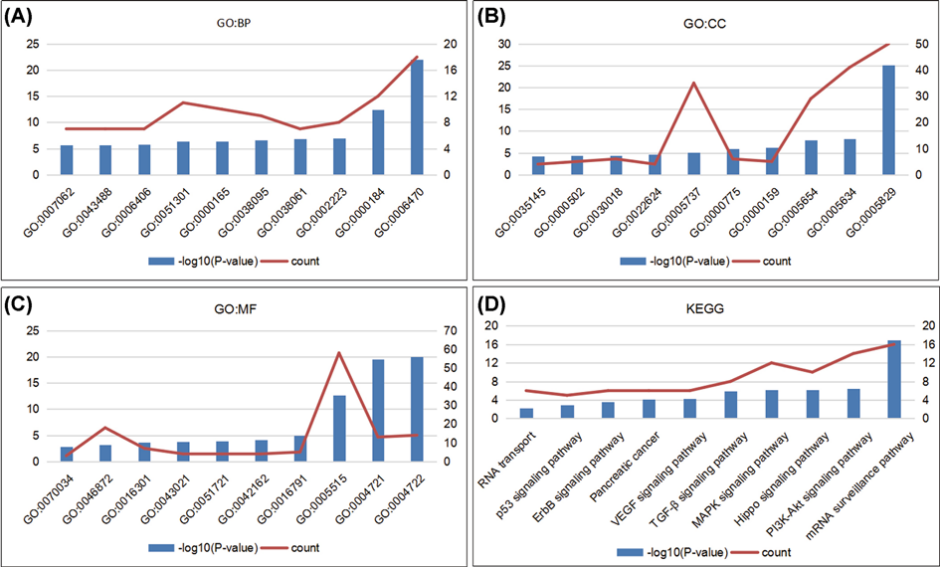
The potential biological function of PPPcs and genes significantly correlated with their alterations were predicted by GO enrichment analysis and KEGG analysis in DAVID Biological processes (**A**), cellular components (**B**) and molecular functions (**C**) of GO enrichment analysis and KEGG analysis (**D**) were used to investigate the potential function of PPPcs and genes significantly correlated with their alterations.

With GO enrichment analysis and KEGG analysis in DAVID, we analyzed the biological role and function of PPPcs and the genes significantly related to them. According to GO enrichment analysis, we found that GO:0006470 (protein dephosphorylation), GO:0000184 (nuclear-transcribed mRNA catabolic process), GO:0006406 (mRNA export from nucleus), and GO:0043488 (regulation of mRNA stability) were significantly affected by alterations in the PPPcs in pancreatic cancer (Figure 4A). In addition, GO:0000159 (protein phosphatase type 2A complex), GO:0022624 (proteasome accessory complex), GO:0043021 (ribonucleoprotein complex binding), and GO:0070034 (telomerase RNA binding) were regulated by alterations in these PPPcs (Figure 4B,C).

KEGG analysis was used to investigate the pathways in which PPPcs and their neighbor genes were involved. Several pathways related to the functions of PPPcs were found through KEGG analysis, and the mRNA surveillance pathway demonstrated the most significant correlation with PPPcs (*P* = 1.05E-17, Figure 4D). Among these pathways, the hsa04151: PI3K-Akt signaling pathway, hsa04010: MAPK signaling pathway, hsa04350: TGF-β signaling pathway, hsa04370: VEGF signaling pathway, hsa04012: ErbB signaling pathway, and hsa04115: p53 signaling pathway correlated with tumorigenesis and pathogenesis of pancreatic cancer (Supplementary Figure S5).

## Discussion

Pancreatic cancer is one of the most lethal malignancies in the world, but it has limited biomarkers and therapeutic targets for its diagnosis and treatment. Currently, compelling evidence suggests that PPPcs, a family of genes encoding the catalytic subunits of PPPs, are involved in a variety of physiological and pathological process, but little is known about PPPcs’ roles in pancreatic cancer. Accordingly, we used bioinformatic analysis to evaluate their expression, prognostic value, and potential function in pancreatic cancer.

PPP1C has three isoforms, PPP1CA, PPP1CB, and PPP1CC, which present with similar substrate specificities. Previous reports have shown that their main functions include glycogen metabolism, gene transcription, cell-cycle progression, and more [11,27]. In addition, PPP1C can bind to the p53 binding protein (p53BP) to form a complex that may regulate the dephosphorylation of p53, a kind of tumor suppressor [28]. More recently, Palam et al. showed that gemcitabine could induce oxidative stress in pancreatic cancer cells and that this process could be reversed by PP1-mediated p-eIF2 dephosphorylation [29]. In this work, we found that the mRNA levels of PPP1CA and PPP1CB in pancreatic adenocarcinoma were higher than those in normal tissues. Furthermore, Kaplan–Meier analysis showed that patients with higher mRNA levels of PPP1CA, PPP1CB, and PPP1CC had poorer prognosis than those with lower levels; such results were also validated at the protein level (*P*<0.01). The network analysis suggested that the pro-tumor effects of PPP1C may contribute to the correlation between PPP1C and TGF-βR2, and a similar mechanism has been found in phytochemical-induced skin collagen biosynthesis [30]. PPP2C, a hotspot among all PPPcs, has two isoforms: PPP2CA and PPP2CB. Accumulating evidence suggests that PPP2C is involved in a variety of biological process, such as apoptosis and metabolism [31]. PPP2, usually considered a tumor suppressor, can regulate important signaling pathways, such as MAPK and Wnt signaling, the deregulation of which can contribute to cancer [32]. Accordingly, Farrell et al. showed that knockdown of a PP2A inhibitor could increase c-Myc degradation and decrease the tumorigenic potential of pancreatic cancer cells [33]. Surprisingly, Oncomine and THPA databases demonstrated no significant difference in either the mRNA or the protein expression of PPP2CA and PPP2CB between pancreatic adenocarcinoma and normal tissues. Conversely, GEPIA revealed that the mRNA expression of them were even higher in pancreatic adenocarcinoma than in normal tissues. In addition, patients with higher mRNA or protein levels of PPP2CA showed poorer prognosis than those with lower levels. Although the difference was not significant, PPP2CB also showed a trend as an unfavorable prognostic factor in THPA. Furthermore, network analysis showed that the genes related to the pathogenesis and progression of pancreatic cancer, such as AKT2 and TP53, would be greatly affected by PPP2CA and PPP2CB alterations. More studies are needed to confirm the expression and pro-tumor function of PPP2C at the molecular, cell, and tissue levels in pancreatic cancer.

In mammals, PPP3CA, PPP3CB, and PPP3CC are the three isoforms of PPP3C, which is known for its role in T-lymphocyte activation and immunosuppression [34]. PPP3CA and PPP3CB are expressed ubiquitously, whereas PPP3CC mainly is expressed in the testis [11]. We found no significant difference in the transcription and protein levels of PPP3CC between pancreatic adenocarcinoma and normal tissues.

Accordingly, PPP3CC was not detected in pancreatic adenocarcinoma or normal tissues by immunohistochemistry. Tahira et al. found that the long noncoding intronic RNA of PPP3CB was differentially expressed in primary and metastatic pancreatic cancer, but its mechanism remained unelucidated [35]. Intriguingly, our work showed that PPP3CA was an unfavorable prognostic factor, but PPP3CB was a favorable prognostic factor for pancreatic cancer. The network analysis revealed a positive link between ERBB2 and PPP3CB, which suggested that PPP3CB might inhibit pancreatic cancer through the ERBB pathway, because the ERBB family are overexpressed in pancreatic cancer and play key roles in its carcinogenesis [36].

PPP4C, PPP5C, and PPP6C are three types of PPPcs without any isoforms, and they are also present in most tissues. Recent evidence suggests an important role of PPP4C in the progression of various cancers [37,38]. Wen et al. found that PPP4C was overexpressed in pancreatic cancer, and its expression correlated with poor prognosis in patients with stage II pancreatic cancer [39]. At the mRNA level, our work showed that PPP4C was the most significantly upregulated gene among all PPPcs in pancreatic cancer with a fold change of 6.153. However, such a significant difference was not observed between pancreatic cancer and normal tissues at the protein level, and PPP4C showed no prognostic value in Kaplan–Meier analysis. PPP5C plays a pivotal role in regulating cell growth, and studies have shown thatPPP5C knockdown could inhibit cancer cell proliferation [40–42]. Zhu et al. found that knockdown of PPP5C could enhance gemcitabine sensitivity by promoting apoptosis of pancreatic cancer cells; and more cells in G0/G1 phase arrest were observed in this condition [43]. In this work, we found that the mRNA expression of PPP5C was higher in pancreatic adenocarcinoma than in normal tissues and that its level correlated with tumor stage. The network analysis suggested a positive link between PPP5C and RAF1, which is a key gene in cell growth control. Among all PPPcs, only PPP6C has been explicitly identified as an oncogene in previous studies; high expression of PPP6C was observed in glioma tissues [44]. Our study demonstrated that the mRNA expression of PPP6C was higher in pancreatic adenocarcinoma than in normal tissues in the GEPIA dataset. However, the survival analysis showed that PPP6C seemed to be a favorable prognostic factor with regard to both transcription and protein levels in pancreatic cancer.

PPP7C, also known as PPEF, has two isoforms: PPEF1 and PPEF2. Ping Ho first showed that a gene variant of PPEF1 (PPEF1V) was highly expressed in T-cell lymphoblastic lymphoma cells [45]. More recently, Soo-Yeon Park et al. found that overexpression of PPEF1 increased chemoresistance and tumorigenic growth of lung cancer cells by suppressing p53-mediated genotoxic stress responses [46]. Kutuzov et al. identified PPEF2as a suppressor for apoptosis signal regulating kinase-1 (ASK1), a MAP kinase implicated in a variety of diseases, including cancer [47]. In addition, recent studies revealed that PPEF2 was crucial to support the survival of immature CD8^+^ dendritic cells, and its down-regulation limited T-cell responses [48]. PPEF1 and PPEF2 showed low expression in both pancreatic adenocarcinoma and normal tissues in this work. However, we found that PPEF2, rather than PPEF1, demonstrated a favorable prognostic value at the mRNA level in pancreatic cancer.

In our study, the expression, prognostic value, and potential function of PPPcs were comprehensively evaluated in pancreatic cancer. Among all the PPPcs, the transcription levels of PPP1CA, PPP1CB, PPP3CA, PPP3CB, and PPP4C were higher in pancreatic cancer than in the normal pancreas. The Kaplan–Meier analysis showed that the high transcription levels of PPP1CA, PPP1CB, PPP1CC, PPP2CA, and PPP3CA correlated with poor survival. In contrast, patients with pancreatic cancer and high transcription levels of PPP3CB, PPP5C, PPP6C, and PPEF2 seemed to have better prognosis. The functional analysis showed that these PPPcs may facilitate anti- or pro-tumor effects of pancreatic cancer through the p53 signaling pathway, ErbB signaling pathway, VEGF signaling pathway, TGF-β signaling pathway, MAPK signaling pathway, and PI3K-Akt signaling pathway. PPP3CC, PPP4C, and PPEF1 demonstrated no prognostic value in the present study, but more studies should be conducted to confirm these results.

## Supplementary Material

Supplementary Figures S1-S5 and Tables S1-S3

## Data Availability

The data used to support the findings of this study are available from the corresponding author Lixia Wu upon request.

## Abbreviations

ASK1: apoptosis signal regulating kinase-1
AUC: area under the curve
BP: biological processes
CC: cellular components
DAB: 3, 3′-diaminobenzidine tetrahydrochloride
GEPIA: Gene Expression Profiling Interactive Analysis
GO: Gene Ontology
KEGG: Kyoto Encyclopedia of Genes and Genomes
MF: molecular functions
OS: overall survival
p53BP: p53 binding protein
p-eIF2: p-eukaryotic initiation factor 2
PPP: phosphoprotein phosphatase
PS: prognostic score
THPA: The Human Protein Atlas
