# Supplementary material for: The role of phosphoprotein phosphatases catalytic subunit genes in pancreatic cancer
Source: Biosci Rep. 2021 Jan 4;41(1):BSR20203282. doi: 10.1042/BSR20203282 (PMC7785039; doi:10.1042/BSR20203282)
Supplement: Supplementary Figures S1-S5 and Tables S1-S3 [file BSR-2020-3282_supp.pdf]

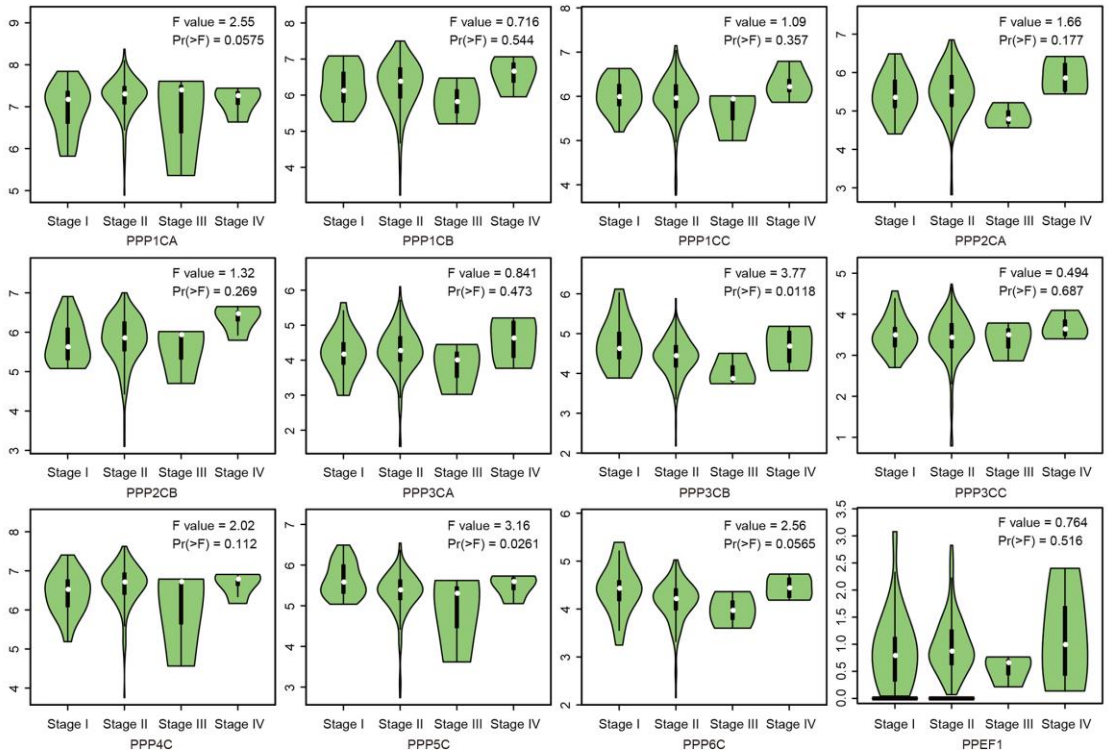

Supplementary Figure 1. The correlations between PPPcs and tumor stage in pancreatic cancer.

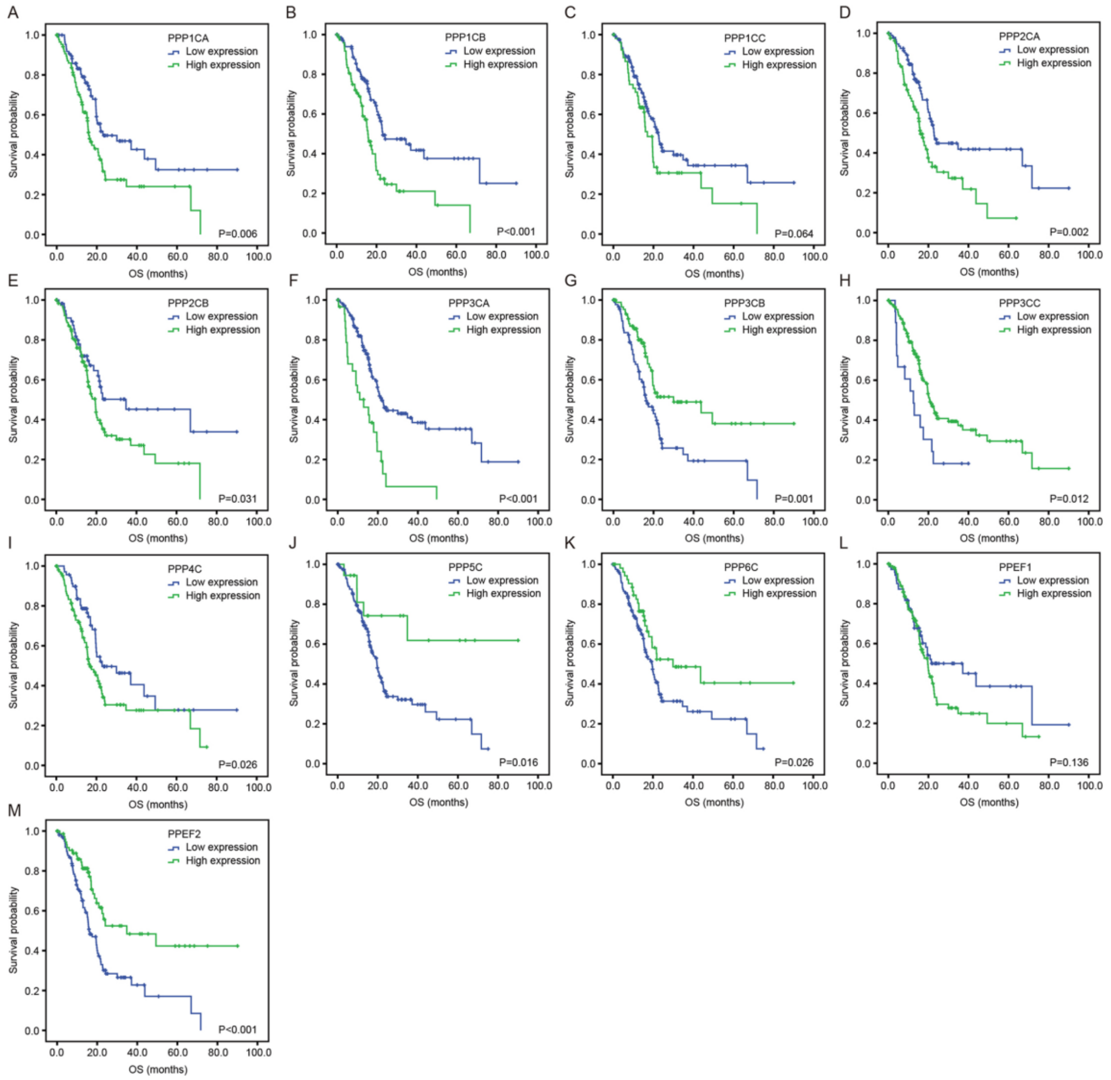

Supplementary Figure 2. Kaplan-Meier analysis estimates OS according to the transcription expression of PPPcs in pancreatic cancer.

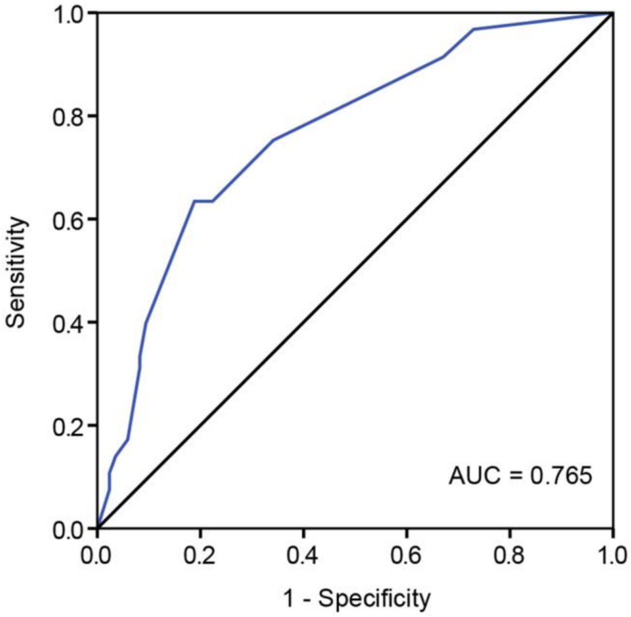

Supplementary Figure 3. Construction of the integrated prognostic classifier of the PPPcs.

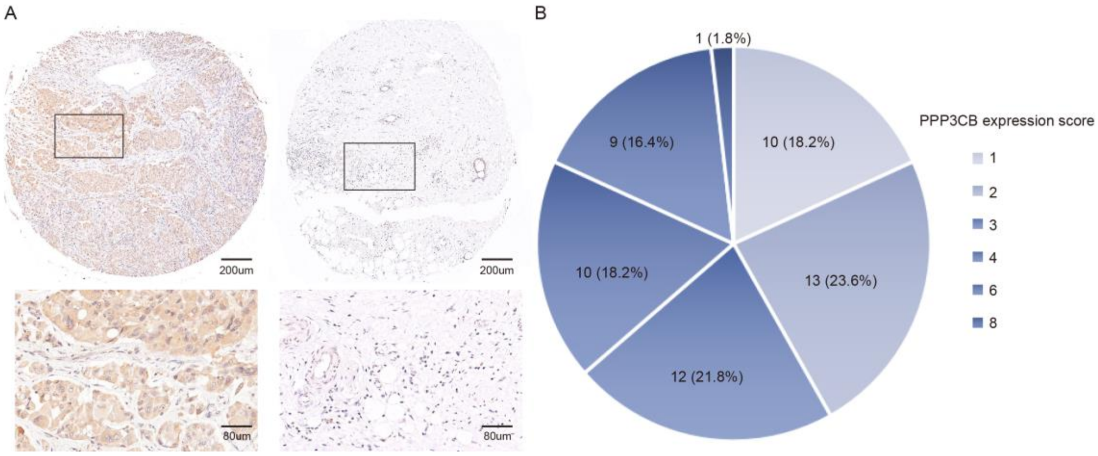

Supplementary Figure 4. The representative images of PPP3CB - high/low expression (A) and the distribution pattern of the immunohistochemical results in pancreatic cancer (B).

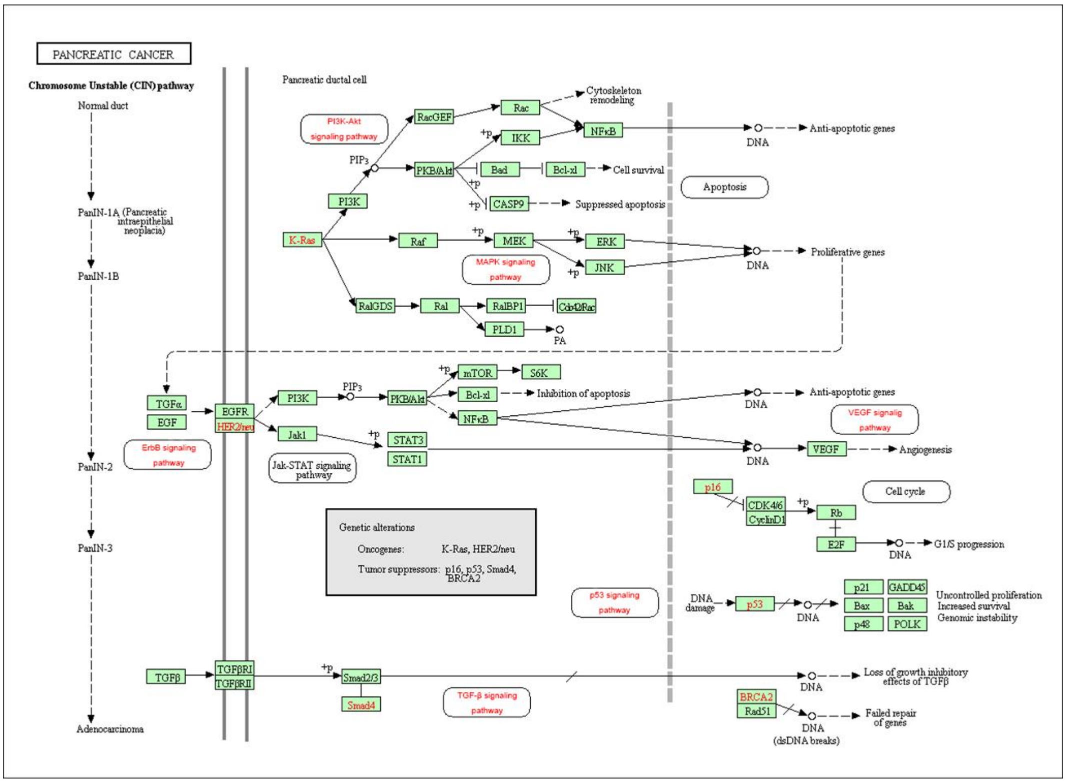

Supplementary Figure 5. Signal pathways affected by PPPcs' alteration in pancreatic cancer (in red).

Supplementary Table 1.Human PPP catalytic subunit genes and their functions

PPPs	PPP catalytic subunits		
	Name	Gene	Reported functions in pancreatic cancer
Ppp1	Ppp1cα	PPP1CA	Regulation of p53, oxidative stress regulation
	Ppp1cβ	PPP1CB	
	Ppp1cγ	PPP1CC	
Ppp2	Ppp2cα	PPP2CA	Promotion of c-Myc degradation, inhibition of TGF-β induced invasiveness
	Ppp2cβ	PPP2CB	
Ppp3	Ppp3cα	PPP3CA	NA
	Ppp3cβ	PPP3CB	
	Ppp3cγ	PPP3CC	
Ppp4	Ppp4c	PPP4C	Overexpression and affecting prognosis
Ppp5	Ppp5c	PPP5C	Promoting apoptosis of cancer cells, cell-cycle regulation
Ppp6	Ppp6c	PPP6C	NA
Ppp7	Ppp7cα	PPEF1	NA
	Ppp7cβ	PPEF2	

Supplementary Table 2. Baseline clinicopathological characteristics of patients with pancreatic cancer

Valuables	Category	Characteristics
Gender	Male	37 (67.3%)
	Female	18 (32.7%)
Age	Median (Range)	63 (36-85)
Primary tumor location	Head and neck	32 (58.2%)
	Body and tail	23 (41.8%)
Nuclear grade	I-II	45 (81.8%)
	III	10 (18.2%)
Vascular invasion	No	28 (50.9%)
	Yes	27 (49.1%)
T stage	1	4 (7.3%)
	2	43 (78.2%)
	3	8 (14.5%)
N stage	0	33 (60.0%)
	1	22 (40.0%)
Jaundice	No	42 (76.4%)
	Yes	13 (23.6%)
Abdominal pain	No	22 (40.0%)
	Yes	33 (60.0%)

Supplementary Table 3. The protein expression level and prognostic value of PPPcs.

Gene	Expression level (number of patients)				Prognostic Value		
	High	Medium	Low	Not detected	Favourable	Unfavourable	Unprognostic
PPP1CA	0	6	3	1		5.05E-03	
PPP1CB	0	10	1	1		1.54E-03	
PPP1CC	1	2	3	5		4.96E-03	
PPP2CA	0	2	1	6		1.34E-02	
PPP2CB	0	2	1	6		4.50E-02	
PPP3CA	0	5	2	4		3.82E-04	
PPP3CB	2	6	2	1	6.96E-05		
PPP3CC	0	0	0	11			8.84E-02
PPP4C	2	6	0	0			2.52E-01
PPP5C	5	5	1	0			5.56E-02
PPP6C	0	2	1	9	1.71E-02		
PPEF1	4	2	1	1			1.70E-01
PPEF2	--	--	--	--			
